# Antenatal Bartter Syndrome: A Review

**DOI:** 10.1155/2012/857136

**Published:** 2012-02-28

**Authors:** Y. Ramesh Bhat, G. Vinayaka, K. Sreelakshmi

**Affiliations:** ^1^Department of Paediatrics, Kasturba Medical College, Manipal University, Udupi District, Manipal 576104, India; ^2^Department of Obstetrics and Gynecology, Kasturba Medical College, Manipal University, Udupi District, Manipal 576104, India

## Abstract

Antenatal Bartter syndrome (ABS) is a rare autosomal recessive renal tubular disorder. The defective chloride transport in the loop of Henle leads to fetal polyuria resulting in severe hydramnios and premature delivery. Early onset, unexplained maternal polyhydramnios often challenges the treating obstetrician. Increasing polyhydramnios without apparent fetal or placental abnormalities should lead to the suspicion of this entity. Biochemical analysis of amniotic fluid is suggested as elevated chloride level is usually diagnostic. Awareness, early recognition, maternal treatment with indomethacin, and amniocentesis allow the pregnancy to continue. Affected neonates are usually born premature, have postnatal polyuria, vomiting, failure to thrive, hypercalciuria, and subsequently nephrocalcinosis. Hypokalemia, metabolic alkalosis, secondary hyperaldosteronism and hyperreninaemia are other characteristic features. Volume depletion due to excessive salt and water loss on long term stimulates renin-angiotensin-aldosterone system resulting in juxtaglomerular hyperplasia. Clinical features and electrolyte abnormalities may also depend on the subtype of the syndrome. Prenatal diagnosis and timely indomethacin administration prevent electrolyte imbalance, restitute normal growth, and improve activity. In this paper, authors present classification, pathophysiology, clinical manifestations, laboratory findings, complications, and prognosis of ABS.

## 1. Introduction

Bartter syndrome is a rare renal tubulopathy first described by Frederic Bartter in 1962. The primary pathogenic mechanism is defective transepithelial chloride reabsorption in thick ascending limb of loop of Henle (TALH). The disease is characterized by hypokalemia, metabolic alkalosis, and secondary hyperaldosteronism with normal to low blood pressure due to renal loss of sodium and hyperplasia of juxtaglomerular apparatus [1, 2]. There are two distinct presentations of Bartter syndrome, namely; antenatal Bartter syndrome (ABS) and classical Bartter syndrome. ABS is the severe form having onset in utero. The awareness of the condition is important for early recognition. The typical features include fetal polyuria, early onset maternal polyhydramnios, intrauterine growth restriction, preterm birth, postnatal polyuria, episodes of dehydration, recurrent vomiting, and failure to thrive [3, 4]. Another syndrome, Gitelman syndrome, is often called as variant of Bartter syndrome. This is a rare autosomal recessive disorder characterized by late onset hypokalemic metabolic alkalosis, hypocalciuria, and hypomagnesemia. History of maternal hydramnios or prematurity will be absent. They are frequently asymptomatic. Muscular weakness and tetany may be present sometimes. Polyuria and growth retardation are not major manifestations. Plasma renin and aldosterone are increased but not to the degree seen in Bartter syndrome. Urinary prostaglandins are not increased.

## 2. Classification and Inheritance of Bartter Syndrome

Antenatal Bartter syndrome has four variants [5, 6] with mild differences in phenotype and genotype (Table 1). Principal clinical features in most of them include early onset polyhydramnios, failure to thrive, prematurity, and nephrocalcinosis. Types I, II, and III have severe antenatal symptoms, prematurity, and failure to thrive, while type IV is a mild salt losing nephropathy with mild antenatal symptoms. Type IV involves chloride channels which are present in distal nephron as well as in inner ear resulting in sensorineural hearing loss in addition. Table 1 shows the new pharmacology based classification with details of the types and molecules affected in each of them. This classification is designed on Bartter syndrome for easy understanding as students and young physicians are more familiar with pharmacologic actions of diuretics at each level of nephron [6].

## 3. Pathophysiology

**Figure 1:**
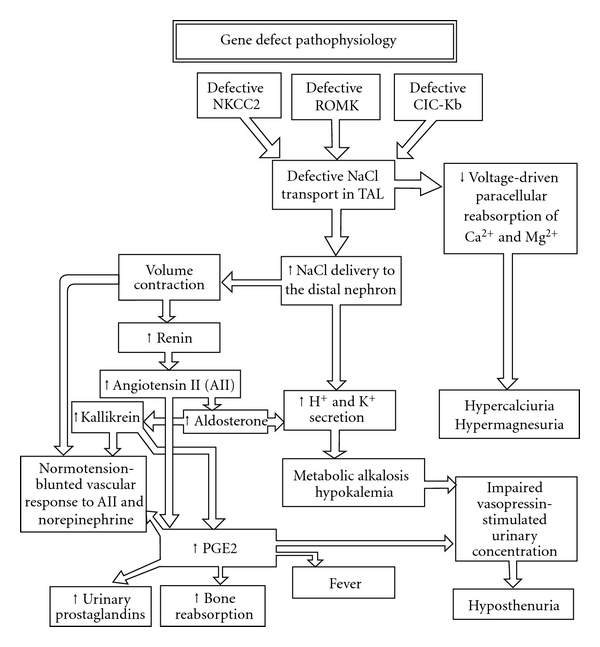
Pathophysiology of Bartter syndrome.

**Figure 2:**
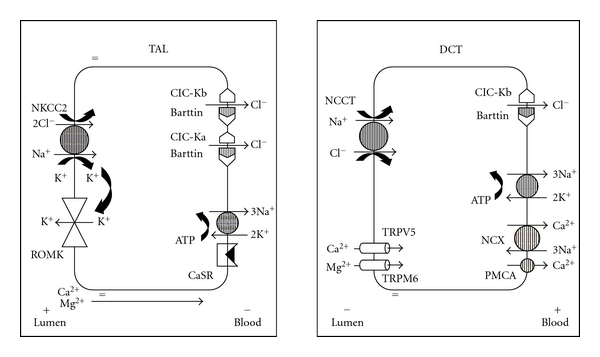
Pictorial representation of ion transporters in thick ascending limb of Loop of Henle (TAL) and distal convoluted tubule (DCT).

Thick ascending loop of Henle (TAL) has channels, namely, Na-K-2Cl cotransporter, K^+^ (ROMK: rat outer medulla potassium), and chloride (CIC-Kb) channels which are responsible for electrolyte absorption. Each of these channels is coded by a specific gene (Table 1). Any mutation in gene results in impaired channel function and hence defective electrolyte reabsorption. K^+^ transport occurs through ROMK channel, whereas Na^+^ and Cl^−^ get absorbed from the luminal space. Passage of Cl^−^ from the cell into the interstitium can take place through kidney-specific chloride channels (CIC-Kb) and via K^+^/Cl^−^ cotransport system. In the apical membrane, there is also an exchange of Na^+^/H^+^. Thus, the handling of chloride ions by the thick ascending loop of Henle (TALH) is an intimate part of the normal function of Na^+^ K^+^ 2CI^−^ electroneutral cotransport, as well as K^+^ channels (ROMK) and Cl^−^ channels (CIC-Kb). Any loss or altered function of Na^+^-K^+^-2CI^−^ cotransporter and/or K^+^ channels as well as chloride channels results in defective Cl^−^ transport. This defect will result in malreabsorption of Na^+^, K^+^, Cl^−^, and Ca^2+^ in the TALH and delivery of large volumes of urine with a high content of Na^+^, K^+^, Cl^−^, and Ca^2+^ to the distal tubule. In the distal tubule, part of the delivered Na^+^ will be reabsorbed in exchange for intracellular K^+^. Hence, potassium wasting occurs. Impaired Na absorption in TALH will result in increased levels of prostaglandin E2. Increased PGE2 will exacerbate primary defect of chloride transport in TALH which will stimulate renin angiotensin-aldosterone axis causing hypokalemia (due to hyperaldosteronism), and impede water reabsorption in collecting ducts leading to hyposthenuria (Figures 1 and 2). Hyperaldosteronism increases K wasting and stimulates exchange of intracellular H ions for K ions for intraluminal K (distal tubule and collecting duct) resulting in exaggeration of metabolic alkalosis. The normal blood pressure despite high levels of renin and angiotensin is thought to be due to nonresponsive of their blood vessels to angiotensins [1–7]. Continuous loss of calcium in urine results in nephrocalcinosis [2–4].

## 4. Clinical Features

Mothers of fetus with Bartter syndrome often present with unexplained polyhydramnios between 24 and 30 weeks of gestation [3, 4, 7]. Intrauterine growth restriction may also be associated. Inability of the kidney tubule to retain salt and water results in fetal polyuria. Important biochemical abnormality in amniotic fluid is normal sodium and potassium but consistently elevated chloride levels [4, 8–12]. Infants are usually born preterm. After birth, important diagnostic finding is hyposthenuria and rapid weight loss. Poor feeding and lethargy are the other symptoms. Urine examination shows low specific gravity, normal potassium but high sodium and chloride levels. However, after 1–3 weeks, level of potassium considerably rises above normal with relatively less sodium than in the first week of life. Prostaglandin levels are high in blood and urine as a secondary phenomenon [5, 6, 9, 13]. Impaired sodium absorption in TALH will result in increased levels of prostaglandin E_2_ [13, 14]. If the diagnosis gets delayed, infants may present with poor feeding, dehydration, and severe electrolyte imbalance. Transient hyperkalemia may be observed in type II ABS. Blood pressure is usually normal. Growth faltering, dwarfism, polydipsia, and weakness may be present in older children. Mild mental retardation is reported in few patients. Facial features such as triangular face, prominent forehead, large eyes, protruding ears, and drooping mouth may be present [15, 16]. Sensorineural deafness is seen in type IV Bartter syndrome. Strabismus, convulsions, and increased susceptibility to infections are also reported [15, 16]. Urinary electrolytes except potassium in second trimester are low in mother's urine in cases of Bartter syndrome [17].

## 5. Laboratory Investigations

When there is early onset unexplained maternal polyhydramnios, ultrasonography should be performed to confirm structurally normal fetus and placenta. If ABS is strongly suspected, one should do amniocentesis and subject amniotic fluid for biochemical analysis. High chloride in amniotic fluid is a consistent finding and diagnostic of ABS [4, 9, 17]. Other electrolytes in the amniotic fluid will be normal. In affected neonates, serum and urinary electrolyte estimation is important. Urinary electrolytes show increased sodium, potassium, and chloride levels. Hypokalemia is the usually observed serum electrolyte abnormality. Blood gas analysis detects metabolic alkalosis. Plasma renin will be usually high. Ultrasonography of the kidneys detects bilateral medullary nephrocalcinosis which is observed after several weeks of severe hypercalciuria [2, 4, 11]. Mutational analysis of the genomic DNA will identify the fundamental defect [5].

## 6. Complications

The important complications of ABS include hypercalciuria leading to nephrocalcinosis [2, 4, 11, 12] and growth restriction. Sensorineural deafness is associated with Bartter syndrome IV. Defects in the barttin subunit of the ClC-Ka and CIC-Kb channels are responsible for sensorineural deafness [15]. Very rarely progressive renal disease, renal failure, and interstitial nephritis can occur. Acute renal failure from rhabdomyolysis due to hypokalemia has also been reported.

## 7. Treatment

Prenatal diagnosis can be made by the high chloride content of the amniotic fluid [18–20] and mutational analysis of genomic DNA extracted from cultured amniocytes obtained by amniocentesis [21]. Once ABS is confirmed, mother should be treated antenatally at the earliest with indomethacin (1 mg/kg/day) in two divided doses [22]. Indomethacin inhibits prostaglandin synthetase, decreases renal salt wasting, reduces fetal urine output, and thereby controls polyhydramnios. Indomethacin may lead to constriction of ductus arteriosus. Hence, patency of ductus arteriosus needs to be monitored in all such fetuses. Rapidly increasing hydramnios may require therapeutic amniocentesis. Indomethacin therapy and therapeutic amniocentesis usually allow the pregnancy to continue. Following birth, neonate should be monitored for urine output, hydration, weight loss, and electrolyte balance. Correction of dehydration and electrolyte imbalance are the important aspects of management. Potassium supplements are usually needed by 2-3 weeks. Prostaglandin synthetase inhibitors are usually required for the disease control. Indomethacin at a dose of 1–5 mg/kg is usually recommended and well tolerated [18, 22, 23]. Early initiation of indomethacin may be required in neonatal Bartter syndrome caused by mutations at gene coding for the NKCC2 transporter. Benefit from initiation of indomethacin therapy at 4–6 weeks and doses below 1 mg/kg/day is likely in patients with mutations at the ROMK channel gene [19]. Indomethacin has side-effects on gastrointestinal tract. Colonic perforation after indomethacin administration has been reported emphasizing the importance of careful monitoring [24]. Other drugs used are acetylsalicylic acid (100 mg/kg/day), ibuprofen (30 mg/kg/day), or ketoprofen (20 mg/kg/day). Addition of potassium sparing diuretics may be initially effective in the control of hypokalemia, but their effect is transient. Caution in such treatment is required as treatment with potassium sparing diuretics may be dangerous in situations of gross salt and water wasting and circulatory volume contraction. Long-term prognosis is guarded. Lack of satisfactory control may lead to morbidity, growth failure, and renal insufficiency [2, 18, 23].

## 8. Prognosis

Untreated ABS patients may succumb to dehydration, dyselectrolytemia, and intercurrent infections. Timely and appropriate therapy results in clinical improvement and catch up growth in majority of children. Long-term outcome including mental development and puberty is usually normal [2, 18, 23]. Growth retardation is a uniform feature in nearly all patients with Bartter syndrome. Developmental delay has also been described in earlier reports. Hypokalemia, hypercalciuria, and nephrocalcinosis may lead to chronic tubulointerstitial nephropathy and progressive reduction in GFR. Renal failure is likely to occur especially in children with BSND mutations. Renal failure requiring dialysis or transplantation is fairly uncommon in Bartter syndrome. Brochard et al. [25] reported chronic renal failure in 3 out of 42 children with a median followup of 8.3 years. Satisfactory prognosis after a median followup of more than 10 years and gallstones representing a new complication of ABS has also been reported [26]. Benefits from renal transplantation have been mentioned. Spontaneous recovery of ABS following a period of treatment has been recognized [27].

## Figures and Tables

**Table 1:** Pharmacological classification of Bartter syndrome with important clinical features.

Subtypes	Gene loci	Molecule affected	Molecule implicated	Site in renal tubule	Pharmacological classification	Important clinical features
Antenatal Bartter syndrome	I (601678)	*SLC12A1*/15q21.1	Na-K-2Cl cotransporter	TAL	Pure frusemide type	Severe maternal polyhydramnios, hypercalciuria, nephrocalcinosis
Antenatal Bartter syndrome	II (241200)	*KCNJ1*/11q24	Kir1.1 potassium channel	TAL	Thiazide type	Hypochloremia, hypomagnesemia, failure to thrive in infancy, EAST syndrome
Classic Bartter	III (602522)	*CLCNKB*/1p36	ClC-Kb chloride channel	DCT	Thiazide type	Hypomagnesemia, hypocalciuria, EAST syndrome
Bartter syndrome with senosorineural deafness	IV (606412)	*BSND*/1p31 or *CLCNKA*- *CLCNKB*/1p36	Barttin, ClC-Ka and ClC-Kb chloride channels	TAL+DCT	Thiazide-frusemide type	Polyuria, hypochloremia, mild hypomagnesemia, SND, CRF

TAL: thick ascending loop of Henle, TAL: thin ascending loop of Henle, DCT: distal cortical tubule, EAST syndrome: epilepsy, ataxia, sensorineural deafness, tubulopathy, SND: sensorineural deafness, and CRF: chronic renal failure.
